# SARS-COV-2 infection in pregnant women and newborns in a Spanish cohort (GESNEO-COVID) during the first wave

**DOI:** 10.1186/s12884-021-03784-8

**Published:** 2021-04-26

**Authors:** Itzíar Carrasco, Mar Muñoz-Chapuli, Sara Vigil-Vázquez, David Aguilera-Alonso, Concepción Hernández, César Sánchez-Sánchez, Cristina Oliver, Mónica Riaza, Marta Pareja, Olga Sanz, Beatriz Pérez-Seoane, Juan López, Elena Márquez, Sara Domínguez-Rodríguez, Alicia Hernanz-Lobo, Juan Antonio De León-Luis, Manuel Sánchez-Luna, María Luisa Navarro

**Affiliations:** 1 Paediatric Infectious Disease Unit, Instituto de Investigación Sanitaria Gregorio Marañón, Madrid, Spain; 2 Facultad de Medicina Universidad Complutense de Madrid, Madrid, Spain; 3 Department of Obstetrics and Gynecology, Hospital General Universitario Gregorio Marañón, Madrid, Spain; 4 Department of Neonatology, Hospital General Universitario Gregorio Marañón, Madrid, Spain; 5 Department of Paediatric Infectious Diseases, Hospital General Universitario Gregorio Marañón, Madrid, Spain; 6 Department of Paediatric Gastroenterology Diseases, Hospital General Universitario Gregorio Marañón, Madrid, Spain; 7 Department of Paediatrics, Hospital Universitario HM Montepríncipe, Madrid, Spain; 8 Department of Paediatrics, Hospital General de Albace, Castilla La Mancha, Spain; 9 Department of Obstetrics and Gynecology, Complejo Hospitalario de Navarra, Navarra, Spain; 10 Department of Neonatology, Hospital Universitario Infanta Sofía, Madrid, Spain; 11 Department of Paediatrics, Hospital Universitario Infanta Leonor, Madrid, Spain; 12 Department of Paediatrics, Hospital San Pedro de Alcántara, Cáceres, Spain; 13 Paediatric Infectious Diseases Unit, Fundación para la Investigación Biomédica del Hospital 12 de Octubre, Madrid, Spain

**Keywords:** COVID-19, Caesarean section, Morbidity, Mortality, Newborn, Pneumonia, Pregnancy, Preterm birth, SARS-CoV-2

## Abstract

**Background:**

Knowledge about SARS-CoV-2 infection in pregnancy and newborns is scarce. The objective of this study is to analyse clinical and epidemiological characteristics of a cohort of women infected with SARS-CoV-2 during pregnancy and their newborns exposed to SARS-CoV-2 during gestation.

**Methods:**

Multicentric observational study of Spanish hospitals from the GESNEO-COVD cohort, participants in RECLIP (Spanish Network of Paediatric Clinical Assays). Women with confirmed SARS-CoV-2 infection by PCR and/or serology during pregnancy, diagnosed and delivering during the period 15/03/2020–31/07/2020 were included. Epidemiological, clinical, and analytical data was collected.

**Results:**

A total of 105 pregnant women with a median of 34.1 years old (IQR: 28.8–37.1) and 107 newborns were included. Globally, almost 65% of pregnant women had some COVID-19 symptoms and more than 43% were treated for SARS-COV-2. Overall, 30.8% of pregnant women had pneumonia and 5 (4.8%) women were admitted to the intensive care unit needing invasive mechanical ventilation. There was a rate of 36.2% of caesarean sections, which was associated with pneumonia during pregnancy (OR: 4.203, CI 95%: 1.473–11.995) and lower gestational age at delivery (OR: 0.724, CI 95%: 0.578–0.906). The prevalence of preterm birth was 20.6% and prematurity was associated with pneumonia during gestation (OR: 6.970, CI95%: 2.340–22.750) and having a positive SARS-CoV-2 PCR at delivery (OR: 6.520, CI95%: 1.840–31.790). All nasopharyngeal PCR in newborns were negative at birth and one positivized at 15 days of life. Two newborns died, one due to causes related to prematurity and another of unexpected sudden death during early skin-to-skin contact after delivery.

**Conclusions:**

Although vertical transmission has not been reported in this cohort, the prognosis of newborns could be worsened by SARS-CoV-2 infection during pregnancy as COVID-19 pneumonia increased the risk of caesarean section deliveries and preterm births.

## Introduction

Severe acute respiratory syndrome coronavirus 2 (SARS-CoV-2) is a novel beta coronavirus that causes coronavirus disease 2019 (COVID-19), a severe infectious respiratory disease. SARS-CoV-2 first case was reported on December 31, 2019, in Wuhan (China) and has since spread worldwide. The World Health Organization declared an International Emergency on January 30 [1], and a global pandemic on March 11th, 2020 [2]. First SARS-CoV-2 confirmed case in Spain was declared on January 31st, 2020. Up to October 22nd, 2020, 40,652,097 cases of SARS-CoV-2 infection have been confirmed and 1,122,036 SARS-CoV-2 related deaths have been reported worldwide (Word Health Organization).

The anatomical, physiological and immunological changes accompanying pregnancy may raise pregnant women’s susceptibility to viral pathogens and increase the risk of developing severe pneumonia [3, 4] . Evidence from other coronavirus infections, such as SARS-CoV or MERS-CoV, suggests that infected pregnant women might be more susceptible to adverse outcomes, including intubation, intensive care unit (ICU) admission, renal failure and death [5]. Hantoushzadeh et al. suggested that pregnant women SARS-CoV-2 infected during the second or third trimester of their pregnancy may experience clinical complications and die [6].

There is insufficient data available to evaluate the effects of SARS-CoV-2 infection on pregnant women and pregnancy outcomes and the prevalence of perinatal complications is still unknown. It has been reported that the most common symptoms of SARS-CoV-2 infection in women during pregnancy are fever, cough, dyspnoea, fatigue and myalgia [7], similar to most frequent symptoms in other adults [8]. The severity of the infection in adults is associated with certain risk factors such as hypertension, obesity, chronic lung disease, diabetes mellitus and cardiovascular diseases [9]. Sentilhes et al. [10] have found that maternal age, obesity, hypertension or diabetes may increase the risk of pregnant women with COVID-19 morbidity, but these factors are not described in detail in the published series of pregnant women. The rate of severe pneumonia in pregnant women has been reported in a wide range from 0 to 14% [11].

The possibility of vertical and perinatal transmission is still controversial [12]. There are some studies testing newborns from women infected during pregnancy at birth by RT-PCR (Real-Time Polymerase Chain Reaction) [13–16], other publications have reported several cases of SARS-CoV-2 infected newborns [17–22] and potential mechanisms of perinatal transmission have been reviewed [12]. SARS-CoV-2 has been found in the placenta by RT-PCR [23, 24] and by electron microscopy [25] but newborns in those studies were not infected. The vaginal fluid has also been tested by RT-PCR, without SARS-CoV-2 detection [26]. Salvatore et al. [27] support that if correct hygiene precautions are taken perinatal infection is unlikely to occur. Intrauterine and peripartum transmission risks from mother to child are unknown and there is no clear evidence on mode and timing of delivery [28]. Little is known about the consequences of SARS-CoV-2 infection in newborns. A case of late-onset infection in a newborn has been reported, suggesting the need to follow up newborns from mothers infected during delivery [19]. It has been suggested that the strong innate immunity of children leads to better control of infection at the entry site [29].

The main objective of the study is to describe clinical and analytical characteristics of SARS-CoV-2 infection in a cohort of infected pregnant women during the first wave of the pandemic. Secondary objectives are the analysis of delivery outcomes and perinatal and postnatal transmission of SARS-CoV-2 infection.

## Methods

A prospective, multicentric, observational study from Spanish GESNEO-COVID cohort. Inclusion criteria were: i) women with confirmed SARS-CoV-2 infection by a positive RT-PCR result during pregnancy on a respiratory specimen (nasopharyngeal swab), by the detection of IgG or/and IgM antibodies in serum or by high clinical suspicions even if RT-PCR or/and antibodies in serum were negative; ii) delivery during 15/03/2020–31/07/2020; iii) inclusion of their newborns. Exclusion criteria were women with miscarriage, foetal death and dead newborns.

The first wave of the pandemic in our cohort comprehended from March 15th 2020 to July 31st 2020. During the first month of the study, due to the diagnostic indications and the scarcity of available tests at the hospitals, only symptomatic women were screened for the infection. As the pandemic was going forward, in many hospitals pregnant women admitted for delivery were tested for SARS-CoV-2 infection through a screening program both by active infection (RT-PCR in nasopharyngeal swab) and past infection (IgG antibodies in serum). Thus, asymptomatic women testing positive for active or past SARS-CoV-2 infection at delivery after April 15th were also included in the study.

Preterm birth was defined as newborn birth at less than 37 weeks of gestation. Newborns were considered small for their gestational age (SGA) when their weigh was equal or less than 10th percentile according to Intergrowth and Fenton criteria. If the maternal clinical condition was acceptable/good, newborns were not separated from their mothers after birth and maternal breastfeeding was recommended [30]. Newborns were tested for SARS-CoV-2 by RT-PCR on nasopharyngeal swabs at 24 h and 15 days of life. Chest X-ray was performed when indicated by the clinical situation and after informed consent. Data was retrieved from each hospital’s electronic medical history and was collected at birth using REDCap (Research Electronic Data Capture) system [31], hosted on a secure server at the Gregorio Marañón Research Institute in Madrid. Socio-demographic, clinical, analytical and virological data from infected women at diagnosis were collected. Obstetric data and delivery outcomes from infected women and characteristics and clinical conditions from newborns were collected.

The study was approved by the Ethics Committee from Hospital Gregorio Marañón and patients informed consent were obtained before inclusion.

### Statistical analyses

Continuous variables were presented with median and interquartile ranges (IQR). Categorical variables were presented as total counts and percentages (%). Chi-square test or Fisher test, as appropriate, were performed to compare categorical variables and U Mann-Whitney test to compare continuous variables. We assessed the association of clinical, laboratory and demographic variables with gestational pneumonia, caesarean and prematurity using multivariable analysis. Variable selection for the models was done by a backwards stepwise procedure using Akaike index criterion. A *p*-value less than 0.05 was considered statistically significant. All analyses were conducted in IBM-SPSS Statistics Version 25.0 (Armonk, NY: IBM Corp) and R Software.

## Results

A total of 105 infected pregnant women and 107 newborns (two twin births) were included from GESNEO-COVID cohort.

### Description of SARS-CoV-2 infected women during pregnancy

The median age of pregnant women at delivery was 34.1 (IQR: 28.8–37.1) years. More detailed characteristics are shown in Table 1. More than half (59.6%) were Caucasian and 28.8% of them were from Latin America. No woman was diagnosed during the first trimester, 6.7% of the women were diagnosed during the second trimester of pregnancy and 93.3% during the third trimester. There were 51.9% SARS-CoV-2 RT-PCR positive tests at delivery. Overall, 34.3% of women had some comorbidity: 6.7% were obese, 1.9% presented hypertension, 1.9% asthma, 6.7% gestational diabetes, 10.5% gestational hypothyroidism, 1.9% immunosuppression and 10.5% presented other comorbidities. No pre-eclampsia was reported.

**Table 1:** Characteristics of women infected with SARS-CoV-2 during pregnancy by pneumonia

	Pregnant women	No pneumonia	Pneumonia	***p-***value*	OR
	***N = 105***^a^	***N = 72***	***N = 32***		
**Age (years)**	34.1 (28.8–37.1)	34.3 [28.5;37.2]	34.0 [31.8;36.4]	0.778	1.01 [0.94;1.08]
**Ethnics**				0.669	
**Caucasian**	63 (60.%)	44 (61.1%)	18 (56.2%)		
**African**	2 (1.9%)	2 (2.78%)	0 (0.00%)		
**Arabic**	1 (1%)	1 (1.39%)	0 (0.00%)		
**LA**	30 (28.6%)	19 (26.4%)	11 (34.4%)		
**GA at diagnosis (w)**	36.9 (33.4–39.2)	38.2 [35.5;39.8]	34.3 [28.2;36.4]	< 0.001	0.83 [0.75;0.92]
**Time of diagnosis**				0.027	
**Second trimester**	7 (6.7%)	2 (2.78%)	5 (15.6%)		Ref.
**Third trimester**	98 (93.3%)	70 (97.2%)	27 (84.4%)		0.16 [0.02;0.85]
**Blood group**				0.640	
**O**	46 (43.8%)	32 (44.4%)	13 (40.6%)		Ref.
**A**	42 (40.0%)	29 (40.3%)	13 (40.6%)		1.1 [0.43;2.78]
**B**	10 (9.5%)	5 (6.94%)	5 (15.6%)		2.46 [0.59;10.3]
**AB**	5 (4.8%)	4 (5.56%)	1 (3.12%)		0.61 [0.03;4.67]
**Rh Positive**	91 (86.7%)	64 (88.9%)	27 (84.4%)	0.314	0.51 [0.14;1.96]
**Any comorbidity**	36 (34.3%)	20 (27.8%)	15 (46.9%)	0.093	2.27 [0.95;5.48]
**Symptomatology**	68 (64.8%)	35 (48.6%)	32 (100%)	< 0.001	
**Pneumonia COVID-19**	32 (30.8%)				
**Treatment**	46 (43.8%)	14 (19.4%)	32 (100%)	< 0.001	
**Need of ICU admission**	5 (4.8%)	0 (0.00%)	5 (15.6%)	0.002	
**Days at ICU**	10.0 [6.5–18.5]		10.0 [6.5–18.5]		
**RT-PCR+ at delivery**	65 (61.9%)	45 (62.5%)	19 (59.4%)	0.933	0.88 [0.37;2.10]
**Caesarean section**	38 (36.2%)	18 (25.0%)	20 (62.5%)	0.004	4.88 [2.02;12.4]
**GA at delivery (w)**	39.0 [37.4;40.0]	39.2 [38.4;40.1]	38.4 [34.1;39.9]	0.006	0.74 [0.62;0.89]
**Preterm birth**	21 (20.6%)	8 (11.6%)	13 (40.6%)	0.002	5.08 [1.85;14.9]

*LA* Latin American, *GA* Gestational Age, *w* weeks, *ICU* Intensive Care Unit, *RT-PCR* Real Time- Polymerase Chain Reaction, *w* weeks. ^a^It was not possible to determine pneumonia or no pneumonia in one pregnant women. *p-value was calculated using Chi-squared or Fisher test for categorical variables and U-Mann Whitney for continuous variables

### Effects of SARS-CoV-2 infection during pregnancy in pregnant women and delivery outcomes

Two thirds (64.8%) of SARS-CoV-2 infected pregnant women had COVID-19 symptoms. The most common symptoms were fever (36.2%), cough (35.2%), and dyspnoea (19.0%). Less frequent symptoms included myalgia (13.3%), anosmia (12.4%), headache (9.5%), rhinorrhoea (8.6%), vomiting (6.7%), diarrhoea (5.7%) and other symptoms (10.5%).

**Fig. 1:**
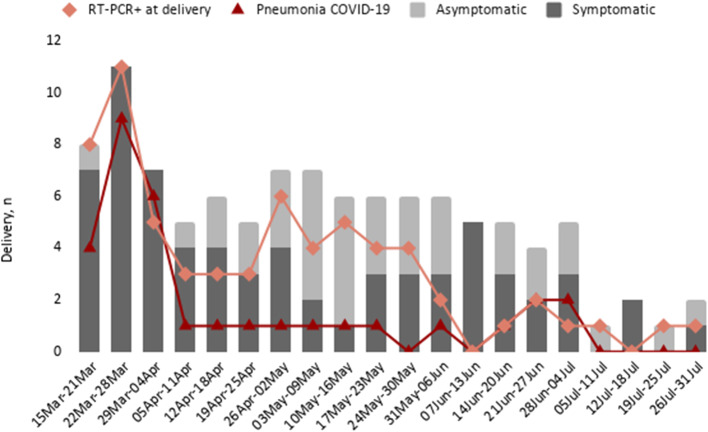
Positive RT-PCR, pneumonia and symptomatology in pregnant women at delivery during the study period (15 March 2020–31 July 2020)

Chest X-ray was performed in 52 women and 32 (61.5%) of them had signs compatible with pneumonia. Overall, radiological images suggested bilateral pneumonia in 20 women and unilateral pneumonia in 12. All 32 pneumonia cases were diagnosed during the first month of the study (15 May-15 April 2020) (Fig. 1). Five women with severe pneumonia and positive RT-PCR at delivery required admission to the Intensive Care Unit for a median of 10.0 (IQR: 6.5–18.5) days, requiring invasive mechanical ventilation. There was no maternal mortality reported.

**Fig. 2:**
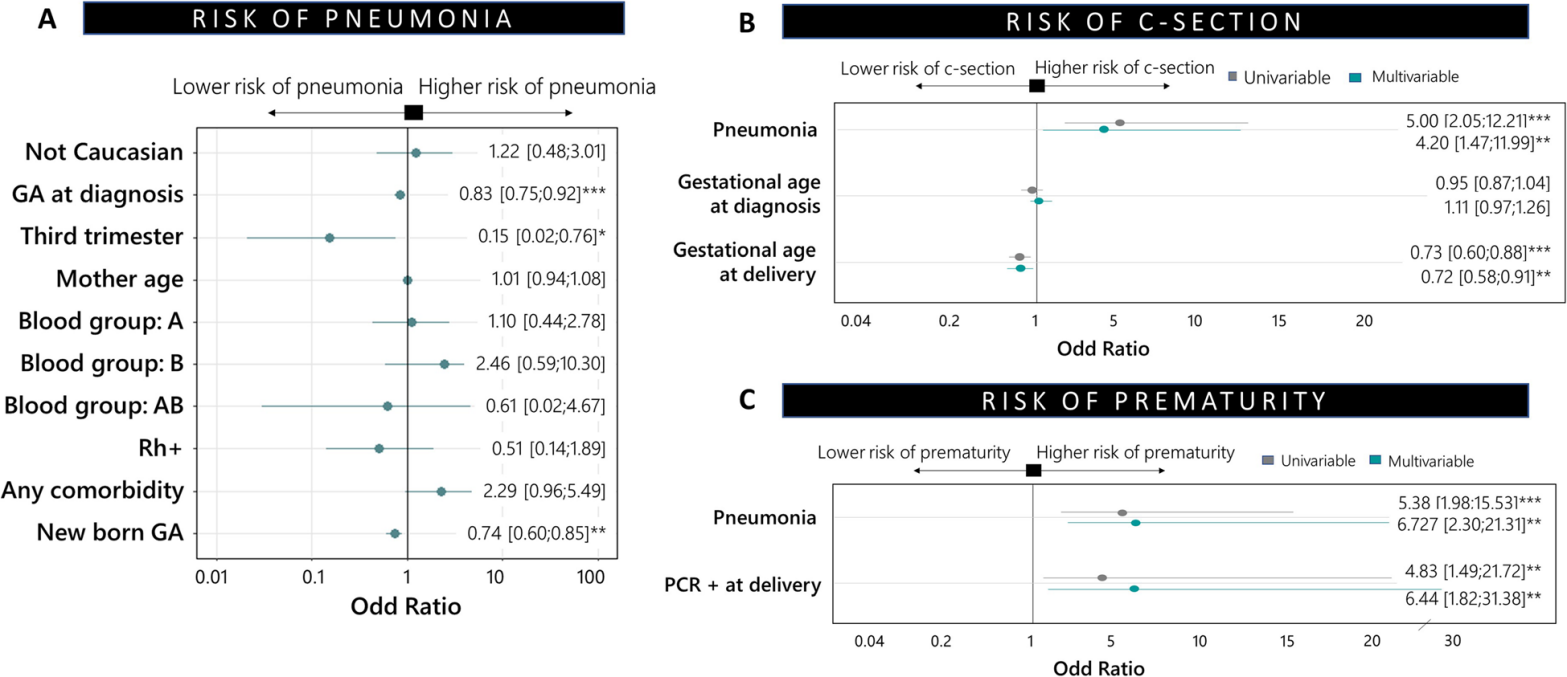
Factors associated with pneumonia during pregnancy, caesarean section at delivery and prematurity: **a** Pneumonia, **b** Caesarean section and **c** Prematurity. * < 0.05; ** < 0.01; *** < 0.001

In the laboratory analyses increased concentrations of D dimer, higher values of liver enzyme alanine aminotransferase and lower count of lymphocytes were found in women with pneumonia compared with women who did not develop pneumonia (Table 2). Obesity was also associated with a higher risk of pneumonia. Lymphopenia (< 1500 lymphocytes/ml) and a lower gestational age at diagnosis were potential predictors for pneumonia in the multivariable analysis (Fig. 2).

**Table 2:** Summary of analytic findings by groups of pneumonia COVID-19

	Pregnant women	No pneumonia	Pneumonia	***p-***value*	OR
	***N = 105***^a^	***N = 72***	***N = 32***		
**Glucose (mg/dL)**	86.0 [78.0;99.0]	80.0 [76.0;88.0]	94.0 [84.8;116]	0.013	1.00 [0.99;1.01]
**Sodium (mmol/L)**	137 [135;138]	137 [136;139]	136 [134;137]	0.014	0.80 [0.64;1.00]
**Potassium (mmol/L)**	4.00 [3.77;4.14]	4.10 [3.90;4.20]	3.85 [3.60;4.07]	0.008	0.10 [0.02;0.62]
**Calcium (mg/dL)**	8.35 [8.20;8.47]	8.40 [8.20;8.40]	8.30 [8.20;8.50]	1.000	1.01 [0.66;1.53]
**AST (U/L)**	54.0 [21.5;155]	23.5 [18.2;151]	78.0 [34.0;146]	0.118	1.00 [1.00;1.01]
**ALT (U/L)**	19.0 [13.0;40.0]	16.0 [12.0;25.0]	24.0 [16.0;52.5]	0.038	1.00 [1.00;1.01]
**GGT (U/L)**	13.5 [8.00;28.8]	13.0 [9.50;24.2]	18.0 [7.75;32.0]	0.710	1.01 [0.99;1.03]
**Urea (mg/dL)**	16.0 [12.8;19.0]	16.0 [13.0;18.0]	16.0 [13.2;19.0]	0.691	1.09 [0.86;1.38]
**Creatinine (mg/dL)**	0.59 [0.49;0.68]	0.60 [0.52;0.66]	0.56 [0.48;0.70]	0.963	2.40 [0.06;92.8]
**LDH (U/L)**	210 [177;270]	212 [170;266]	209 [182;284]	0.677	1.00 [1.00;1.00]
**CPK (units/L)**	47.0 [31.0;113]	47.0 [37.0;113]	49.0 [26.5;99.2]	0.469	1.00 [0.98;1.01]
**CRP (mg/dL)**	4.60 [1.75;8.86]	4.00 [1.30;9.20]	5.00 [2.15;8.57]	0.930	1.00 [1.00;1.01]
**Ferritin (mcg/L)**	47.0 [30.0;76.0]	38.0 [22.2;48.5]	73.0 [42.5;238]	0.101	1.00 [1.00;1.01]
**D-dimer (ng/mL)**	637 [438;1325]	864 [616;1844]	493 [404;660]	0.011	1.00 [1.00;1.00]
**Nt-proBNP (ng/L)**	269 [163;1128]		269 [163;1128]	.	
**Fibrinogen (mg/dL)**	629 [560;694]	610 [567;676]	642 [551;712]	0.518	1.00 [1.00;1.01]
**Troponin (ng/L)**	1.60 [1.60;2.40]	1.60 [1.60;2.80]	1.60 [1.60;2.15]	0.718	1.01 [0.88;1.17]
**Procalcitonin (mcg/L)**	0.06 [0.02;0.10]	0.03 [0.02;0.06]	0.06 [0.04;0.10]	0.072	1447 [0.01;172,837,716]
**Platelets (cells/mm**^**3**^**)**	1300 [900;1800]	1600 [1200;2100]	950 [725;1200]	< 0.001	1.00 [1.00;1.00]
**Haemoglobin (g/dL)**	198,000 [162,250;240,250]	204,000 [164,500;234,500]	193,000 [146,250;261,000]	0.909	1.00 [1.00;1.00]

*AST* Aspartate Aminotransferase, *ALT* Alanine Aminotransferase, *GGT* Gamma Glutamyltransferase, *LDH* Lactate Dehydrogenase, *CPK* Creatinine Phosphokinase, *CRP* C Reactive Protein, *nT-proBNP* N-terminal pro-B-type Natriuretic Peptide. ^a^*It was not possible to determine pneumonia or no pneumonia in one pregnant women.* *p-overall was calculated using U-Mann Whitney for continuous variables

Overall, 46 women (43.8%) received treatment under study for COVID-19. Different treatment options were used and sometimes combined; antiviral treatment: lopinavir/ritonavir (28 women), gamma interferon (4 women) and remdesivir (2 women); anti-malarial: hydroxychloroquine (33 women); antibiotic treatment: ceftriaxone (25 women) and other antibiotics (16 women); immunomodulatory drugs: systemic corticoids (8 women) and tocilizumab (4 women); and other treatments (13 women). 61.5% of women received treatment before delivery and 38.5% received treatment the day of delivery or after, including both women receiving remdesivir. Treatments were safe for both mother and newborns.

Globally, 38 (36.2%) of pregnant women had a caesarean delivery and the indication for caesarean section was severe COVID-19 for 28.9% of them. Pregnant women with pneumonia had a 5-fold increased risk of caesarean sections and premature newborns than those without pneumonia in the univariable analysis (OR:5.0 [2.0;12.2], *p*-value < 0.001) (Table 3). Pneumonia and lower gestational age at delivery were associated with caesarean section as predictor factors in the multivariable analysis (Fig. 2). The women with pneumonia were 4 times at greater risk than those without pneumonia (OR:4.2[1.47–11.99], *p* = 0.007) adjusted for gestational age at diagnosis and delivery. All women admitted to the ICU had caesarean sections due to the severity of the disease.

**Table 3:** Characteristics of women exposed to SARS-CoV-2 during gestation by c-section

	Pregnant women	No C-section	C-section	***p-***value*	OR
	***N = 105***	***N = 67***	***N = 32***		
**Age (years)**	34.1 [28.9;37.0]	34.9 [30.9;36.9]	33.1 [27.8;36.9]	0.233	0.95 [0.89;1.02]
**Ethnics**				0.766	
**Caucasian**	63 (65.6%)	41 (68.3%)	22 (61.1%)		Ref.
**African**	2 (2.08%)	1 (1.67%)	1 (2.78%)		
**Arabic**	1 (1.04%)	1 (1.67%)	0 (0.00%)		
**LA**	30 (31.2%)	17 (28.3%)	13 (36.1%)		
**GA at diagnosis (w)**	36.9 [33.6;39.1]	37.6 [34.2;39.4]	36.6 [31.7;39.0]	0.325	0.95 [0.87;1.04]
**Time of diagnosis**				0.096	
**Second trimester**	7 (6.67%)	2 (2.99%)	5 (13.2%)		Ref.
**Third trimester**	98 (93.3%)	65 (97.0%)	33 (86.8%)		0.21 [0.03;1.10]
**Blood group**				0.488	
**O**	46 (44.7%)	31 (47.0%)	15 (40.5%)		Ref.
**A**	42 (40.8%)	28 (42.4%)	14 (37.8%)		1.03 [0.42;2.55]
**B**	10 (9.71%)	5 (7.58%)	5 (13.5%)		2.04 [0.48;8.71]
**AB**	5 (4.85%)	2 (3.03%)	3 (8.11%)		2.96 [0.41;27.7]
**Rh Positive**	91 (88.3%)	59 (89.4%)	32 (86.5%)	0.752	0.76 [0.22;2.82]
**Any comorbidity**	36 (34.3%)	24 (35.8%)	12 (31.6%)	0.821	2.27 [0.95;5.48]
**Symptomatology**	68 (64.8%)	38 (56.7%)	30 (78.9%)	0.038	2.81 [1.15;7.48]
**Pneumonia COVID-19**	32 (30.8%)	12 (18.2%)	20 (52.6%)	< 0.001	4.88 [2.02;12.4]
**Treatment**	59 (56.2%)	43 (64.2%)	16 (42.1%)	0.047	0.41 [0.18;0.93]
**Need of UCI admission**	5 (4.76%)	1 (1.49%)	4 (10.5%)	0.056	6.94 [0.92;195]
**Days at ICU**	10.0 [10.0;14.0]	10.0 [10.0;10.0]	12.0 [8.25;16.2]	0.717	1.07 [0.74;1.54]
**RT-PCR+ at delivery**	65 (61.9%)	39 (58.2%)	26 (68.4%)	0.409	1.54 [0.67;3.68]
**GA at delivery (w)**	39.0 [37.6;40.0]	39.4 [38.4;40.1]	38.1 [34.6;39.9]	0.003	0.73 [0.60;0.88]
**Preterm birth**	21 (20.6%)	7 (10.9%)	14 (36.8%)	0.004	4.62 [1.69;13.8]

*C-section* Caesarean section, *LA* Latin American, *GA* Gestational Age, *w* weeks, *ICU* Intensive Care Unit, *RT-PCR* Real Time- Polymerase Chain Reaction, *w* weeks. *p-value was calculated using Chi-squared or Fisher test for categorical variables and U-Mann Whitney for continuous variables

### Effects of SARS-CoV-2 during pregnancy in newborns

Overall, 53.3% of newborns were women and median weight at delivery was 3.050 kg (IQR: 2780–3455) with 5.6% of newborns were small for gestational age (Table 4). Median gestational age at delivery was 39.0 (IQR: 37.6–40.0) weeks with a range from 24 to 41 weeks. Apgar score at 1 and 5 min ranged from 1 to 9 with a median of 9.0 (IQR: 9.0–9.0) and 3 to 10 with a median of 10.0 (IQR: 10.0–10.0). Prematurity rate was 20.2%. From all newborns, 16.8% required admission to a neonatal intensive care unit with a median duration of 3.0 (IQR: 1.0–8.0) days. None of the newborns presented any symptom of SARS-CoV-2 infection. The most common complications were due to prematurity. 66.4% of the newborns were breastfed.

**Table 4:** Characteristics of newborns exposed to SARS-CoV-2 during gestation by prematurity

	Newborns^a^	No preterm	Preterm	***p-***value*	OR
	***N = 107***	***N = 72***	***N = 32***		
**Men**	47 (46.7%)	44 (53.0%)	12 (57.1%)	0.968	0.87 [0.32;2.31]
**Breastfeed**				0.003	
**Maternal**	71 (66.4%)	59 (72.8%)	9 (42.9%)		Ref.
**Artificial**	18 (16.8%)	9 (11.1%)	9 (42.9%)		6.33 [1.97;21.2]
**Mixed**	16 (15.0%)	13 (16.0%)	3 (14.3%)		1.54 [0.29;6.21]
**Apgar 1’**	9.00 [9.00;9.00]	9.00 [9.00;9.00]	9.00 [8.00;9.00]	0.054	0.63 [0.39;1.02]
**Apgar 5’**	10.0 [10.0;10.0]	10.0 [10.0;10.0]	10.0 [9.00;10.0]	0.009	0.52 [0.28;0.98]
**Small for GA**	6 (5.61%)	5 (6.02%)	1 (4.76%)	1.000	0.86 [0.03;6.05]
**Symptomatology**	81 (75.7%)	69 (83.1%)	9 (42.9%)	< 0.001	0.16 [0.05;0.44]
**Need of NICU Admission**	18 (16.8%)	6 (7.23%)	12 (57.1%)	< 0.001	17.6 [5.36;65.4]
**Days at NICU**	3.00 [1.00;8.00]	1.00 [0.50;1.50]	6.50 [2.75;14.8]	0.015	1.61 [0.91;2.85]
**Oxygen need**	13 (12.1%)	4 (4.82%)	9 (42.9%)	0.203	3.44 [0.54;26.6]
**Days of oxygen**	6.00 [1.00;19.0]	5.50 [1.00;10.8]	9.00 [1.00;29.0]	0.470	1.05 [0.95;1.16]
**Surfactant need**	6 (5.61%)	1 (1.20%)	5 (23.8%)	0.333	3.70 [0.40;118]
**RT-PCR at birth**				0.344	
**Negative**	101 (94.4%)	77 (92.8%)	21 (100%)		Ref.
**Not realized**	6 (5.61%)	6 (7.23%)	0 (0.00%)		
**RT-PCR at 15 days**				0.700	
**Negative**	60 (56.1%)	44 (53.0%)	13 (61.9%)	0.700	Ref.
**Positive**	1 (0.93%)	1 (1.20%)	0 (0.00%)		

*D* Days, *GA* Gestational age, *RT-PCR* Real Time- Polymerase Chain Reaction*.* ^a^Gestational age information of three newborns was not available. *p-value was calculated using Chi-squared or Fisher test for categorical variables and U-Mann Whitney for continuous variables

An extreme premature newborn (24 week) died at 20 days of life due to prematurity-related complications. Another full-term newborn died during the first 24 h from delivery, due to Sudden infant death Syndrome. Both newborns were born from women with severe pneumonia and admitted at the ICU.

Potential predictors for prematurity were diagnosed during the second trimester of pregnancy, having a positive RT-PCR at delivery, pneumonia during pregnancy and caesarean delivery in the univariable analysis. Having a positive RT-PCR at delivery and COVID-19 pneumonia during pregnancy were predictor factors for prematurity in the multivariable analysis (Fig. 2).

### Perinatal transmission of SARS-CoV-2 infection

No vertical transmission was detected. Nasopharyngeal RT-PCR was possible to perform at 24–48 h of life in 101 newborns and repeated at 15 days old. None newborn tested positive at birth and one of the 61 (1.6%) neonates tested at 15 days of life had a positive result with a previous negative result at birth. This case was a full-term newborn whose mother tested positive for SARS-CoV-2 RT-PCR during the 24 h previous to admission. Delivery was by caesarean section due to worsened maternal diseases, and after delivery, the mother was admitted at the ICU with COVID-19 pneumonia. The newborn was discharged after testing negative for SARS-CoV-2 RT-PCR at birth and lived with her asymptomatic grandmother, after the 15 days’ diagnosis the neonate was tested positive for 2 SARS-CoV-2 RT-PCR for 2 weeks and under telephonic follow-up. That child was not breastfed, so this case was considered an intra-family transmission.

## Discussion

SARS-CoV-2 infection during pregnancy could cause COVID-19 pneumonia, that could condition an alteration during the pregnancy. In our series, pregnant women with COVID-19 pneumonia had a higher risk of caesarean sections (*p* = 0.007; OR = 4.20, IC95%: 1.47–11.99) and premature newborns (*p* = 0.002; OR = 6.97, IC95% 2.34–22.75) than those not developing pneumonia.

COVID-19 pneumonia has been associated with gestational age at diagnosis, the older gestational age, the lower risk of pneumonia, for each week that increases gestational age, there is a 21% lower risk of pneumonia. It has also been associated with lymphopenia, having lymphopenia is 11 times more risk of pneumonia than not having it. The rate of severe pneumonia in pregnant women with SARS-CoV-2 infection in our study was 33%, increasing the rate reported in a review from Juan et al.^7^ (0–14%). The high rate of pneumonia diagnosed in this cohort could be explained because only patients with the moderate-severe disease were diagnosed and asymptomatic pregnant women delivering during the first month were not included at the beginning of the pandemic. Laboratory findings were consistent with values reported in non-pregnant adults with SARS-CoV-2 infection including elevated inflammatory index such as C-reactive protein and fibrinogen.

We report a high rate of caesarean sections with a higher risk in pregnant women with pneumonia (4 times more risk). In our results, we find a 36.2% of caesarean section deliveries, close to 41.5% reported by Khoury et al. [32], which is increased compared with the latest upload of caesarean sections data in Spain (26.6%) during 2015 (Instituto Nacional de Estadística, INE base, 2015). The rate of preterm births has been 20.6%, increased compared to 14.6% reported by Khoury et al. [32] and definitively increased compared with the Spanish rate in 2015 (8.18%) (Instituto Nacional de Estadística, INE base, 2015). This data supports Sentilhes et al. [10] and Li N et al. [33] reporting a higher rate of preterm delivery in infected women compared with non-infected women. In our study women developing pneumonia had 7 times more risk of preterm birth than women not developing pneumonia.

Elevated liver enzymes and D-dimer results have been found in women with pneumonia, as it was described before in other Spanish hospitals [11] but in our series, it has not been associated with pneumonia. Treatments used were safe for pregnant women and their newborns. All pregnant women diagnosed with SARS-CoV-2 received heparin for ten to fourteen days by protocol and there were no thromboembolic complications.

Vertical transmission has not been objective and a horizontal transmission case was detected in this study. Even positivity on RT-PCR testing, the newborn did not present any symptomatology at the diagnosis nor during the follow-up. As Buonsenso et al. [19] have reported previously, we report a case of late onset infection in a 15 days old baby born from a woman infected during pregnancy. Maternal breastfeeding has been discarded as a source of transmission because the mother was admitted at the ICU with severe pneumonia, so it has been speculated an intra-family transmission. SARS-CoV-2 could be transmitted to newborns by close contact when not using appropriate hygiene measures [34]. Our study reinforces the national and international recommendations based on not modifying the type of delivery, not separating the mother from the newborn at birth and promoting breastfeeding as well as recommending to maximize hygiene by performing isolation of the contact in the environment.

Our results suggest that gestational age at diagnosis is associated with developing pneumonia, so it could be recommended to implement SARS-CoV-2 infection screening during pregnancy.

All treatment options received by infected women were safe for both women and newborns. Remdesivir has not been used during pregnancy in our cohort, both women receiving remdesivir was after delivery.

Maternal breastfeeding was indicated to infected women in our cohort following national recommendations and we have not detected breastfeeding transmission. The case of the newborn infected horizontally at home highlight the need to follow hygienically measures with newborns at home to avoid intrafamiliar infection. Additional research is required about clinical implications of SARS-CoV-2 infection during pregnancy.

There is a high research gap in SARS-CoV-2 infection during pregnancy. It would be interesting to elucidate if there are potential long-term effects in women infected during pregnancy or possible consequences in newborns exposed to the virus during gestation. There is controversial data regarding the severity of SARS-CoV-2 infection during pregnancy; in our cohort, there is no mortality. Some studies have reported similar outcomes in infected pregnant women compared to non-pregnant adults with COVID-19 [35], but others have reported an increase in morbidity [36].

There are still unanswered questions, for example, what are the implications for women infected during the first trimester? Is there any consequence for newborns exposed to SARS-CoV-2 in a long term? These are important issues to clarify.

The most relevant limitation of our study is that at the beginning of the pandemic, the criteria for testing in Spain, and later by the neighbouring countries, only included patients with significant disease, which implicated the loss of asymptomatic infected women at delivery during the first month. All this, taking into account this is the largest multicentre study analysing both mother and newborn exposed to SARS-CoV-2 infection characteristics and outcomes and following newborns after birth.

## Conclusions

As a conclusion, SARS-CoV-2 infection when symptomatic in pregnant women originated from admissions to the hospital during gestation and increased the time of admission of those with symptoms at delivery, women with pneumonia admitted to the ICU were at risk of maternal death. Even though the vertical transmission has not been reported in our cohort, the prognosis of newborns could be worsened by SARS-CoV-2 infection during pregnancy as we have seen COVID-19 pneumonia increases caesarean section deliveries and preterm births, increasing morbidity and mortality risks for women and newborns.

## Data Availability

The datasets used and/or analysed during the current study are available from the corresponding author on reasonable request.

## Abbreviations

SARS-CoV-2: Severe acute respiratory syndrome coronavirus 2
COVID-19: Coronavirus disease 2019
ICU: Intensive care unit
RT-PCR: Real Time Polymerase Chain Reaction
SGA: Small for gestational age
IQR: Interquartile ranges, OR = Odds ratio
